# Tracheostomy Duration Is a Key Predictor of Persistent Tracheocutaneous Fistulas in Children

**DOI:** 10.7759/cureus.78539

**Published:** 2025-02-05

**Authors:** Patrícia S Sousa, Gil Coutinho, Pedro Alexandre, Diogo Pereira, Jorge Spratley, Carla P Moura

**Affiliations:** 1 Department of Otorhinolaryngology, Unidade Local de Saúde São João, Porto, PRT; 2 Department of Otorhinolaryngology, Unidade Local de Saúde Trás-os-Montes e Alto Douro, Vila Real, PRT; 3 Department of Surgery and Physiology, Faculty of Medicine, University of Porto, Porto, PRT; 4 CINTESIS (Centro de Investigação em Tecnologias e Serviços de Saúde; Research Center for Health Technologies and Services), Faculty of Medicine, University of Porto, Porto, PRT; 5 Department of Medical Genetics, Faculty of Medicine, University of Porto, Porto, PRT; 6 RISE (Rede de Investigação em Saúde; Health Research Network) – Health, University of Porto, Porto, PRT

**Keywords:** pediatric, pediatric tracheostomy, post tracheostomy, tracheo-cutaneous fistula, tracheostomy decannulation (td), tracheostomy timing

## Abstract

Introduction

Pediatric tracheostomy is a surgical procedure that secures the airway in children with upper airway obstruction or those in need of pulmonary support. Persistent tracheocutaneous fistula is a potential sequela that can occur after the decannulation process.

Objectives

To evaluate the frequency and risk factors that lead to a persistent tracheocutaneous fistula.

Material and methods

This is a retrospective analysis of pediatric patients who underwent tracheostomy decannulation in a tertiary hospital from 2008 to 2021.

Results

Nineteen patients, ranging from 6 days to 17 years old, underwent tracheostomy and were later decannulated. Twelve cases (63.2%) closed spontaneously while 7 patients (36.8%) required surgical closure of the tracheocutaneous fistula. Variables such as neurologic comorbidities, laryngotracheomalacia, and prolonged ventilation did not show statistically significant differences between the two groups. The mean tracheostomy duration was 7.8 and 26.7 months for spontaneous and surgical closure of a tracheocutaneous fistula, respectively. Most of the spontaneous closures (83.3%) occurred after 1.5 months. When comparing surgical and spontaneous closure, those needing surgical closure had a tracheostomy for a longer time (p=0.022), especially if the tracheostomy tube was placed more than 12 months (p=0.045).

Conclusion

In this series, most patients had a spontaneous closure of the tracheocutaneous fistula. The main factor for a persistent tracheocutaneous fistula was prolonged tracheostomy, which may be considered an outcome predictor whenever decannulation is planned.

## Introduction

Tracheostomy is a surgical procedure that secures the airway through an artificial opening in the trachea [1]. In children, the main indications include upper airway obstruction, congenital malformations, and conditions requiring pulmonary support or a tracheobronchial toilet such as neuromuscular disorders or chronic lung disease [2-5]. Historically, airway obstruction caused by inflammation or infection of the upper airway was a major indication but has decreased significantly due to vaccination [2,3,5].

A tracheocutaneous fistula (TCF) represents a tract between the trachea and the skin that develops secondary to the decannulation process. In some cases, due to the squamous epithelium lining the tracheostomy tract [6], the stoma site closure may be delayed or prevented, with a negative impact on quality of life, requiring surgical management.

This study aims to evaluate the frequency and risk factors that lead to persistent TCF and to determine the rates of spontaneous and surgical closure.

## Materials and methods

Study design

A retrospective cohort study was conducted in the Department of Otorhinolaryngology at a tertiary university hospital, including pediatric patients who underwent tracheostomy decannulation between January 2008 and December 2021.

Clinical assessment

Children underwent decannulation according to our department’s protocol. The decannulation process begins with a flexible and/or rigid laryngotracheoscopy to assess the indications and safety of decannulation. Following this, the tracheostomy tube is downsized and then capped during the daytime, always under adult supervision and oxygen saturation monitoring. Cases deemed more unstable and prone to respiratory distress are admitted to the hospital to undergo a preliminary full 24-hour surveillance with a capped tracheostomy in the ward, with medical supervision and continuous oxygen saturation monitoring. Children suspected of obstructive sleep apnea undergo a polysomnographic evaluation prior to decannulation. 

Variables

Patients' demographic data, indication for tracheostomy, age at tracheostomy and decannulation, medical comorbidities, ventilation status, tracheostomy duration, and method of closure were evaluated.

Patients with a tracheostomy tube still in place at the time of the study, patients who had undergone surgical closure of a TCF following laryngotracheal reconstruction, and patients with incomplete medical records were excluded from the study.

Statistical analysis

Statistical analysis was performed using the Statistical Package for the Social Sciences (SPSS, version 26.0; IBM Corp., Armonk, NY, US). Frequency analysis and non-parametric tests were used to evaluate the data. A p-value below 0.05 was considered statistically significant.

Ethical considerations

All procedures followed the regulations established by the Ethics and Clinical Research Committee and the Helsinki Declaration of the World Medical Association. Approval for this study was obtained from the Ethics Committee of Unidade Local de Saúde São João (CE-324-23).

## Results

Pediatric tracheostomy was performed in 66 patients, of whom 19 underwent successful decannulation, excluding patients who were successfully decannulated following laryngotracheal reconstruction. Among the patients who were decannulated, 57.9% were male and the mean age at tracheostomy placement was 4.4 ± 6.7 years (median: 0.4 years; range: 6 days to 17 years). Eleven (57.9%) tracheostomies were performed in children under one year of age. The mean age at decannulation was 6.4 ± 6.4 years (median: 3.6 years; range: 19 days to 18 years) and the mean duration of tracheostomy was 14.7 ± 16.4 months (median: 8.0 months; range: 7 days to 48 months).

The main indication for tracheostomy was upper airway obstruction in 68.4% of cases such as laryngotracheal obstruction and craniofacial abnormalities. In 31.6% of cases, the indication was respiratory insufficiency caused by neurological diseases. Most patients had one or more comorbidities such as laryngotracheomalacia (26%), neurological impairment (47%), and prolonged ventilation (58%). The baseline characteristics of the patients are described in Table 1.

**Table 1:** Indications for tracheostomy CNS: central nervous system

Upper airway obstruction (n=13, 68.4%)
Laryngotracheal Obstruction (n=10, 52.6%)
Acquired subglottic stenosis (n=1, 5.26%)
Laryngotracheal fissure (n=1, 5.26%)
Laryngotracheal cleft (n=1, 5.26%)
Laryngeal lymphangioma (n=1, 5.26%)
Vocal fold/laryngeal cyst (n=2, 10.52%)
Supraglottitis (n=1, 5.26%)
Tracheoesophageal fistula (battery ingestion) (n=1, 5.26%)
Acute leukemia (lymphoid infiltration) (n=1, 5.26%)
Nasopharyngeal teratoma (n=1, 5.26%)
Craniofacial Abnormalities (n=3, 15.8%)
Apert syndrome (n=1, 5.26%)
Pierre-Robin syndrome (n=1, 5.26%)
Beckwith-Wideman syndrome (n=1, 5.26%)
Ventilatory support (n=6, 31.6%)
Neurological diseases (n=6, 31.6%)
Brainstem tumor (n=1, 5.26%)
Brain tumor (n=1, 5.26%)
Tetraplegia (CNS infection) (n=1, 5.26%)
Head trauma (n=2, 10.52%)
Steinert myotonic dystrophy (n=1, 5.26%)

When comparing the upper airway obstruction group to the ventilatory support group, no statistical differences were observed regarding the age of tracheostomy (p=0.056, mean of 2.3 and 8.8 years, respectively) or tracheostomy duration (p=0.823, mean of 14.9 and 14.5 months, respectively).

Twelve patients (63.2%) experienced spontaneous closure of TCF, whereas seven patients (36.8%) required surgical closure. The mean tracheostomy duration was 7.8 months for spontaneous closure and 26.7 months for surgical closure. After decannulation, the mean time to spontaneous closure was 1.5 months (range: 7 days to 2.5 months) and the mean time to surgical closure was 9.6 months (range: 3 to 19 months). Most of the spontaneous closures (83.3%) occurred within 1.5 months. Surgical closure was successfully achieved via primary closure, with no reported complications.

There were no statistically significant differences between the spontaneous and surgical closure groups in terms of primary indication for tracheostomy, age at tracheostomy or decannulation, neurologic comorbidities, laryngotracheomalacia, or prolonged ventilation. However, the duration for tracheostomy was statistically significant, with patients requiring surgical closure having a longer duration of tracheostomy (p=0.022), especially in cases where the tracheostomy tube had been in place for more than 12 months (p=0.045) (Table 2).

**Table 2:** Variable analysis between groups of spontaneous closure and surgical closure of tracheocutaneous fistulas ^a^Fisher’s Exact Test; ^b^Mann-Whitney Test

Variables	Spontaneous closure (n=12)	Surgical closure (n=7)	P value
Primary indication for tracheostomy: Airway obstruction (n=13, 68.4%), Ventilatory support (n=6, 31.6%)	n=9, n=3	n=4, n=3	0.617^a^
Age at tracheostomy (mean)	4.5 years	4.3 years	0.932^b^ (U=41)
Age at decannulation (mean)	5.9 years	7.1 years	0.204^b^ (U=27)
Laryngotracheomalacia (n=5, 26%)	n=3	n=2	1.000^a^
Need for prolonged ventilation (n=11, 58%)	n=6	n=5	0.633^a^
Neurological comorbidities (n=9, 47%)	n=5	n=4	0.650^a^
Tracheostomy duration (mean)	7.8 months	26.7 months	0.022^b^ (U=15)
Tracheostomy tube placed more than 12 months (n=7, 36.8%)	n=2	n=5	0.045^a^

## Discussion

The current study indicates that a prolonged tracheostomy is the primary factor contributing to the persistence of TCF.

Pediatric tracheostomy, though relatively rare, is a lifesaving procedure [7] that requires a significant learning curve for caregivers to ensure proper care [8]. Once the underlying condition is resolved, it will eventually result in decannulation and tracheostomy closure.

When proceeding with a decannulation protocol, it is crucial to assess whether the indication for tracheostomy has been resolved and if the conditions for decannulation are met [2,3,9,10]. A stepwise decannulation program, determined by each institution, is mandatory and contributes to the safety and success of the procedure [10,11].

After decannulation, the caregivers are instructed on tracheostomy care, to promote spontaneous closure and minimize complications, such as stoma occlusion, maintenance of a clean and dry stoma, and avoidance of manipulation. In addition, close monitoring in the outpatient clinic is required to assess the TCF closure.

About half of the tracheostomy stoma sites close spontaneously [6,12], with the majority closing within the first month and almost all within four months [6].

The incidence of persistent TCF varies significantly in the literature, with reports up to 57% [1]. The main complications from persistent TCF include draining secretions, skin irritation, inadequate subglottic pressure generation that results in poor cough and recurrent aspiration, altered phonation, poor cosmesis, and persistent risk of water penetration [1,3,6].

Surgical closure is usually performed through fistulectomy followed by multilayered sutures [6], a technique also performed in our department. However, fistulectomy followed by secondary intention closure can be an option [6]. Although both primary and secondary closure techniques appear to be equally effective and associated with acceptable low complication rates [13-15], closure by secondary intention tends to have fewer risks of infection, subcutaneous emphysema [12], and pneumothorax [6] while also reducing hospitalization time and eliminating the need for pediatric intensive care unit monitoring [15]. There is still no consensus regarding the optimal technique for TCF closure and a lack of standardization in perioperative management [16]. In our department, following surgical closure, the patients are monitored overnight and discharged the following day.

The time required for spontaneous closure and the optimal time to consider surgical closure after decannulation are not well established. This study reports a surgical closure time range of 3 to 19 months, while other literature varies from 3.5 to 14 months [6,12]. Some studies suggest considering surgical closure of TCF after an observation period of 6 to 12 months [12,16-18].

Several factors have been connected to the persistence of TCF, though, not all are consistently agreed upon by authors. The current investigation shows that a longer duration of tracheostomy is associated with an increased risk of persistent TCF, in line with previous publications [1,2,6,19]. As such, considering our study’s demographic data, we regard that a surgical TCF closure may be advisable whenever a tracheostomy lasts longer than 12 months. Interestingly, some studies report a critical period of 24 months [1,20,21]. This can be explained by the prolonged duration of mucocutaneous tissue overgrowth and squamous epithelialization in patients with long-term tracheostomies [1].

The primary indication for tracheostomy, whether upper airway obstruction or pulmonary support, does not appear to be associated with persistent TCF [1,6]. Yet, some studies suggest that patients with traumatic injuries or infection [22] or those with maxillofacial and laryngotracheal trauma may achieve decannulation earlier than those with cardiopulmonary and neurological conditions [23], which may influence TCF incidence.

Other factors, such as younger age at tracheostomy [6,17,20] and laryngotracheomalacia [6], have been reported to contribute to persistent TCF, but with limited clinical evidence. In adults, factors like obesity, previous radiation, tracheostomy technique, and previous tracheostomy have been listed as risk factors for persistent TCF [6,19]. However, these factors have not been studied in the pediatric population so far.

Limitations

This is a single-center, retrospective, observational study with a small-sized and heterogenous sample of decannulated patients and, although used in a systematized way by all surgeons, the surgical technique as well as the postoperative management and outcomes after closure were not evaluated.

## Conclusions

The present series shows that the main factor for persistent TCF was prolonged tracheostomy, especially when lasting more than 12 months. This factor could be considered an outcome predictor whenever decannulation is planned and clinicians might lower their threshold for considering surgical closure in such cases. Larger multicenter and prospective studies are needed to determine whether other factors contribute to the persistence of TCF.
